# Epigenetics and cerebral organoids: promising directions in autism spectrum disorders

**DOI:** 10.1038/s41398-017-0062-x

**Published:** 2018-01-10

**Authors:** Sheena Louise Forsberg, Mirolyuba Ilieva, Tanja Maria Michel

**Affiliations:** 1 Department of Psychiatry, Institute for Clinical Research, University of Southern Denmark, Odense, Denmark; 2 Department of Psychiatry, Psychiatry in the region of Southern Denmark, Odense, Denmark; 3 Odense Center for Applied Neuroscience BRIDGE, University of Southern Denmark, Psychiatry in the Region of Southern Denmark, Odense University Hospital, Odense, Denmark

## Abstract

Autism spectrum disorders (ASD) affect 1 in 68 children in the US according to the Centers for Disease Control and Prevention (CDC). It is characterized by impairments in social interactions and communication, restrictive and repetitive patterns of behaviors, and interests. Owing to disease complexity, only a limited number of treatment options are available mainly for children that alleviate but do not cure the debilitating symptoms. Studies confirm a genetic link, but environmental factors, such as medications, toxins, and maternal infection during pregnancy, as well as birth complications also play a role. Some studies indicate a set of candidate genes with different DNA methylation profiles in ASD compared to healthy individuals. Thus epigenetic alterations could help bridging the gene–environment gap in deciphering the underlying neurobiology of autism. However, epigenome-wide association studies (EWAS) have mainly included a very limited number of postmortem brain samples. Hence, cellular models mimicking brain development in vitro will be of great importance to study the critical epigenetic alterations and when they might happen. This review will give an overview of the state of the art concerning knowledge on epigenetic changes in autism and how new, cutting edge expertise based on three-dimensional (3D) stem cell technology models (brain organoids) can contribute in elucidating the multiple aspects of disease mechanisms.

## Introduction

Autism is characterized by a clinical triade of symptoms such as impairment of social interactions and communication as well as restrictive and repetitive patterns of behaviors and interests. Lately, the Diagnostic and Statistical Manual of Mental Disorders 5th edition (American Psychiatric Association, 2013)^1^ has classified infantile autism, Asperger’s syndrome, childhood disintegrative disorder, and pervasive developmental disorders not otherwise specified (PDD-NOS) as autism spectrum disorder (ASD), usually appearing before the age of 3 years. The term “spectrum” denotes a variety of symptoms and severity from mildly afflicted, highly functioning individuals to extremely afflicted individuals in need of lifelong support and care (World Health Organization). Autism is the psychiatric disorder with the highest heritability rate, the concordance rate for monozygotic twins is >90%^2^, and many susceptibility genes have been identified during the past decade. Moreover, there is evidence of environmental factors, such as, e.g., hypoxia during birth, contributing^3^. There is a high prevalence of autistic symptoms in syndromes with chromosomal aberrations, such as Fragile X syndrome and Tuberous Sclerosis^4^. However, even in those disorders following the classical genetic Mendelian rules, a penetrance of <50% has been reported, indicating that epigenetic factors might play an important role in explaining at least part of the neurobiology of autism^5^.

Epigenetic alterations are defined as non-permanent and potentially heritable changes that regulate the expression of genes through alterations to the shape and configuration of DNA, rather than nucleotide sequence. This leads to changes in the ability of certain genes to be transcribed—meaning, that the chromatin threads carrying the genetic information can unwind, coil more tightly, loop, and interact with other proteins to turn certain genes on or off. Epigenetic control can, besides from direct DNA modification^6^, also take the form of modifications in the three dimensional (3D) structure and packaging of DNA, histones, and noncoding RNA-related factors^7^. Chromatin is a dynamic structure. Its accessibility for transcription factors is determined by certain modifications, including DNA methylation as well as acetylation, phosphorylation, ubiquitination, and methylation of histones. They could modify histone proteins, nucleosome movement, and even larger genomic regions. This change in histone modifications has been pointed to as being the gene–environment interface along with DNA methylation^6,8^. In the case of DNA methylation, a methyl group (-CH3) is added to cytosine, leading to gene silencing. Chromatin remodeling could entail sliding of the nucleosome cores by the disassembling/reassembling of the core and could either induce or repress expression. Histone modifications involves amino acids on the terminal end that can bind to methyl, acetyl, phosphate, or ubiquinone, and the most known effect being that of acetylation of lysine in which chromatin is opened and transcription induced. RNA interference is a process that includes RNA silencing complexes that, e.g., bind to mRNA and blocks the ribosome, thereby promoting gene silencing.

**Fig. 1:**
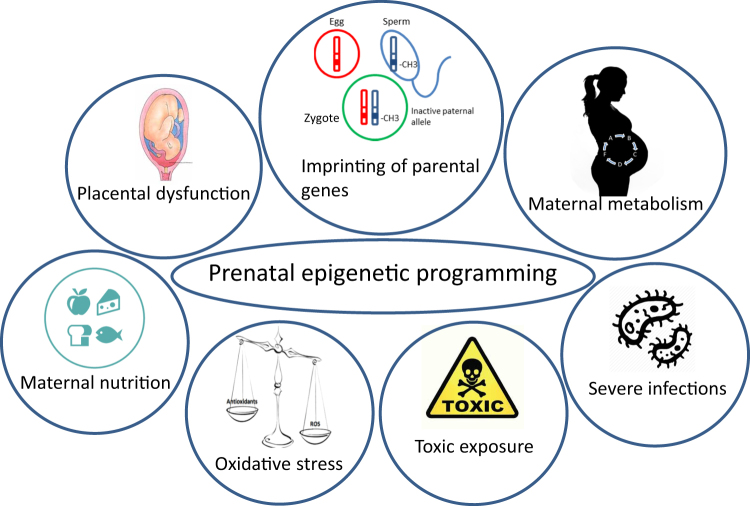
Prenatal epigenetic programming Several prenatal factors could lead to epigenetic dysregulation in ASD. In utero development could be affected by maternal environmental conditions, such as maternal toxin exposure, severe infections, placental dysfunction and hormonal disruptions, oxidative stress, maternal nutrition, and metabolic imbalance. This can result in activation of adaptation mechanisms for survival and growth restriction, which in turn leads to alteration in cellular organization through disturbed proliferation/maturation balance; epigenome modification via DNA methylation, histone modification, and RNA interference; and alteration in cellular metabolic pathways

Epigenetic mechanisms are involved in regulating the prenatal development by directing processes, such as cell proliferation and differentiation, as well as tissue specification^9^. Epigenetic alterations are partly due to environmental factors, which thereby affect the phenotype by modulating gene expression^7^. Since the underlying causes of ASD remain elusive, epigenetic alterations take the role of the environment with regards to gene expression into account. Certain environmental conditions or fluctuations can stimulate epigenetic changes of the genome (Fig. 1). Therefore, epigenetic modulations are a promising candidate to explain the complex neurobiology leading to ASD. Research involving rodents showed that rat pups receiving limited maternal care resulted in the change of the expression of stress-related genes. Those alterations were also maintained in future generations^10^. Similarly, the effect of maternal care on gene expression has also been seen in nonhuman primates^11^. Moreover, research on human subjects has revealed that childhood abuse alters DNA methylation patterns in the brain, resulting in epigenetic changes to the gene expression^12^. Furthermore, a dysregulation of growth factors has been found in a number of adults with ASD. These growth factors are involved in neuronal growth, differentiation, proliferation, and survival in the course of neurodevelopment and can also modulate axonal and dendritic outgrowth^13–18^. DNA methylation of the brain-derived neurotrophic factor (*BDNF*) gene has for instance been linked to early life adversity, including autism^13^. Epigenetic processes and the interaction with environmental conditions might help to explain the differences in gene expression patterns in ASD patients. It is still not entirely clear which environmental factors are responsible for the epigenetic changes seen in ASD, but many have been suggested: environmental toxins, parental age (especially of fathers), maternal diet, and chemical exposure^19–22^. An environmental factor that has been suggested is the maternal use of valproic acid (an anticonvulsant and potent histone deacetylase inhibitor) in early pregnancy^23^. The maternal use of valproic acid during pregnancy could affect the production of GABAergic neurons via the blocking of histone deacetylase^24^. A study also found that severe maternal viral infection in the first trimester of the pregnancy and bacterial infection in the second trimester was associated with a diagnosis of ASD in the child^25^.

Along similar lines to the gene–environment interaction is the investigation of oxidative stress’ role in ASD. One recent study (total *n* = 189) including adults with ASD (*n* = 92) pointed to increased oxidative stress in the patient’s group, in that it found significantly increased levels of superoxide dismutase 1 (although not superoxide dismutase 2 or xanthine oxidase (XO))^26^. Promising results suggest a major role of oxidative stress in the neurobiology of other neurodevelopmental and psychiatric disorders, which are often comorbid with autism^27–32^ such as depression^27,29,30^ and schizophrenia^28,31^. There have been several reports connecting depressive disorders and oxidative stress^27^. The pro-oxidant XO has been studied in this regard and been found to be elevated in both schizophrenia^28^ and depression^29^. Another study investigated whether oxidative stress could contribute to the brain structural changes seen in patients with recurrent depressive disorder^30^. This study, consisting of seven patients and seven healthy controls, then compared manganese (Mn) and copper/zinc (Cu/Zn) superoxide dismutase (SOD) concentrations in postmortem prefrontal cortex and hippocampal brain tissue. The study found that the concentration of Cu/Zn-SOD was increased in the prefrontal cortex although not in the hippocampus of the patients^30^. Another study investigated impaired oxidative stress defense in schizophrenia spectrum disorder by determining the concentrations of Cu, Zn and MnSOD in postmortem brain tissue^31^. This study found significantly elevated levels of Cu, Zn and MnSOD levels in frontal cortex and substantia innominata of the samples^31^.

Although the epigenetic mechanisms involved in autism are not yet fully understood, there are findings suggestive of genome-wide dysregulation and epigenetic alterations in ASD^33^. These studies point to DNA methylation as a likely contributor in the development of the disorder^34^. There are certain syndromes that have been linked to ASD. DNA methylation in connection to imprinting and X-chromosome inactivation could be relevant to the field of ASD research. X-chromosome inactivation is a process in which one of the copies of X chromosomes is inactivated and this is also achieved through DNA methylation. It might be associated with autism, as inactivation or removal of inactivation could lead to genetic aberrations. An example for this is the formation of tiny “X rings” in females. These rings prevent the inactivation of genes within it resulting in an overexpression and could furthermore end in developmental abnormalities such as those seen in certain Turner syndrome cases—a minority with mental retardation and developmental delay^35^. The deficits seen in this syndrome are most commonly related to social reciprocity and communication; in addition to that, it is also associated with an increase in the risk of autism especially in incidences in which the maternally inherited X chromosome was retained^36,37^. The authors surmised that this was due to an X-linked imprinted gene and the expression of which affecting the risk of autism^36^. DNA methylation is also involved in long-distance chromosomal interactions, in which methylation signals interact with other chromosomes or faraway parts of the same chromosome to alter gene expression. This phenomenon can be seen in chromatin looping, in which gene sequences are brought together by the formation of a loop, enabling long-distance gene activation^38^.

Imprinting is a differential inhibition of gene expression (imprinted genes are not expressed) depending on its parental origin. In this way, imprinting controls the expression of either the paternal or the maternal gene in the offspring^39^. Imprinting’s role in ASD has been suggested in several instances^36,37,40,41^, and it is interesting to note that the imprinted domain at 15q11-13 is responsible for Angelman syndrome^42^ as well as Prader–Willi syndrome^43^. In the case of Angelman syndrome, children suffering from the syndrome also tend to be diagnosed with ASD^40,41,44^. One of these studies investigated the prevalence of Angelman Syndrome in school children and analyzed its comorbidity with autistic disorder and found that 4 out of the almost 49,000 conformed to a diagnosis of Angelman syndrome and that all 4 also met full behavioral criteria for a diagnosis of autistic disorder^40^. Another study investigated 23 Angelman syndrome patients and made a comorbid diagnosis of ASD in 14 out of the 23 patients (61%)^41^. Angelman syndrome is associated with a maternal deletion in the chromosomal region 15q11-13 affecting *UBE3A* (related to ubiquitine pathway) expression while the paternal allele is silenced^42^. Prader–Willi syndrome is, on the other hand, caused by the deletion of the parental copies of the *SNRPN* located in the same region (15q11-13). At the same time, due to imprinting the maternally inherited copies are silenced. This is linked to psychotic episodes in the cases of maternal uniparental disomy or imprinting abnormalities^43^. Also worth mentioning is that the 15q11-13 imprinting region has been associated with the increased incidence of autism when maternally duplicated^45^. This study conducted microsatellite and methylation analyses of three children and their parents, in which two of the children had autism and the third did not. It was found that the two individuals had a maternal inheritance of a 15q11-13 duplication while the unaffected child did not have this duplication. Interestingly, a recent study conducted on mice might have discovered the first proof of principle with regards to epigenetic-based therapy, in particular Prader–Willi syndrome^46^. The study found that two selective inhibitors (*UNC0638* and *UNC0648*) activated imprinted genes on the maternal chromosome of cells usually repressed in those with the disorder. Considering this discovery as a possible epigenetic-based treatment of Prader–Willi^46^, imprinting might yet provide some clues to the etiology and possible treatments for ASD as well.

In summary, epigenetic processes are multifaceted and can be connected to multiple genes or genetic pathways. This could make it difficult to identify the essential targets and pathways involved in epigenetic changes. It is believed that epigenetic processes play a major role in brain development and neurodevelopmental disorders^47^. For all the genetic insights that have been gained, we still do not have a full picture of the etiological basis of ASD. It is only recently that more emphasis has been put on environmental factors and this means that epigenetic mechanisms should also be investigated to further ASD knowledge.

## Epigenome-wide analysis and candidate genes in ASD

Several studies have focused on DNA methylation levels on a genome-wide scale (or EWAS) and one very recent study has investigated histone acetylation (see Table 1).

**Table 1:** Epigenome-wide studies and findings

	Study description	Tissue	Participants	Findings
EWAS				
Ginsberg et al.^103^	Genotyping and DNA methylation (DNAm) sequencing	Brain: cerebellar and occipital tissue	9 ASD individuals and 9 controls	Found no changes in DNA methylation between the groups. Found downregulation of genes of MOP and protein translation
Ladd-Acosta et al.^48^	DNAm	Brain: DLPC, temporal cortex and cerebellum	19 autism cases and 21 controls	Found 4 significant DMRs in: *TSPAN32*, *PRRT1*,* ZFP57*, *SDHAP3*
Nardone et al.^49^	DNAm	Brain: nterior cingulate gyrus and prefrontal cortex	ACG: 11 ASD cases and 11 controls	Found 5329 DMPs with significant methylation changes compared to controls. Found brain regions less epigenetically varied than the controls. Implicated genes *HDAC4*and *C11ORF21*
			PFC: 12 ASD cases and 12 controls	
Peripheral tissues				
Nguyen et al.^104^	Global methylation profile of LCLs	Lymphoblastoid cell line	3 pairs of twins discordant in autism	2 candidate genes, *BCL-2* and *RORA*, exhibited hypermethylation of specific CpG sites (gene silencing)
Wang et al.^105^	DNAm in PBCs	Blood	5 ASD children and 5 age/sex-matched controls	Indicated *ENO2* to a subset of autism and pointed to as potential biomarker. This association has not been found since
Wong et al.^50^	Differentially methylated CpG sites of whole blood samples	Blood	50 pairs of monozygotic twins	Genes found to be differentially methylated: *NFYC*, *DUSP2*
				Similarities in *PXDN* and *C11ORF1* in at least two discordant twins
				MDPs in *MGC3207*,* OR2L13*,* FAM181A*
Berko et al.^51^	DNAm	Buccal epithelium	47 ASD cases and 48 controls	Found ASD-specific DMRs. A DMR in the *OR2L13* gene promoter of the ASD-specific DMP also found in peripheral blood
HAWAS				
Sun et al.^106^	Histone acetylation population study	Brain: prefrontal and temporal cortex, cerebellum	45 ASD samples and 49matched controls	More than 68% of syndromic and idiopathic ASD cases share common acetylome signature in the prefrontal and temporal cortex

*DNAm* DNA methylation, *MOP* mitochondrial oxidative phosphorylation, *DLPC* dorsolateral prefrontal cortex, *DMR* differentially methylated region, *ACG* anterior cingulate gyrus, *PFC* prefrontal cortex, *DMP* differential methylated position, *LCL* B-cell derived lymphoblastoid cell lines, *PBC* peripheral blood cell, *HAWAS* histone acetylome-wide association study

These EWAS studies have identified potential biomarkers that could be useful in future studies and implicated certain brain regions with signs of epigenetic dysregulation. Regarding proline-rich transmembrane protein (*PRRT1*), a study found lower methylation of a differentially methylated region (DMR) in the regions of the temporal cortex, cerebellum, and prefrontal cortex^48^. Similarly, a region related to zinc finger protein 52 (*ZFP52*) showed higher methylation of a DMR in the temporal cortex^48^, as well as higher methylation of the cingulate cortex^49^. Tetraspanin 32 gene/chromosome 11 open reading frame 21(*TSPAN32/C11ORF21*) has also been shown to have lower methylation levels in a DMR in the temporal cortex and cerebral cortex^48^, along with lower methylation in the anterior cingulate gyrus and prefrontal cortex^49^. Olfactory receptor family 2 subfamily L member 13 (*OR2L13*) is a special case having shown both significantly increased and decreased methylation in studies^50,51^. Although they have provided insights, there are also some associated issues with the EWAS studies conducted on postmortem brain samples. The most fundamental issue is the uncertainty in how many conclusions can be drawn from postmortem samples to a disorder that arises through the course of development^52^. The dependency on bio-banks also limits the number of samples one can attain. All of the aforementioned studies had fewer than 100 samples and should optimally include more for increased power of the study. There are only a limited number of samples obtainable from bio-banks. Therefore, cell culture systems could offer a viable alternative and will be discussed later on.

Even as genome-wide approaches seem to be the focus in most studies, candidate genes (see Table 2) and their methylation status could still offer valuable insights into the neurobiology of ASD. Although certain genes appear to be involved in the disorder, investigating epigenetic alterations will contribute to get a better understanding of the complex mechanisms involved. Differences in methylation have been observed in different areas of the brain^47,53–59^, and the CpG island (genomic regions >200 bp in length with high CpG sites—in which a cytosine nucleotide is followed by a guanine nucleotide in the linear sequence of bases) known to regulate the oxytocin receptor (*OXTRA*) has presented increases in the DNA methylation status in the temporal cortex as well as in blood (plasma)^47^. Further studies of, e.g., methylation status in blood and other more easily obtained peripheral tissues could possibly result in finding helpful biomarkers for the disorder. The biomarkers could assist the diagnostic process and improve the prognosis, as well as possibly identifying individuals at risk of ASD eventually leading to preventive measures. Other epigenetic mechanisms, aside from DNA methylation itself, have not been investigated nearly as closely and could be involved in the pathogenesis as well.

**Table 2:** Candidate genes and suggested mechanisms

Candidate gene	Function of the gene	Possible epigenetic mechanisms
*OXTR*	G-protein coupled receptor for oxytocin. Modulates: stress, anxiety, social memory, maternal–offspring behavior, etc.	Hypermethylation and silencing^47^
		Decreased *OXTR* expression
*GABRB3*	Responsible for a protein that is a part of the gamma-aminobutyric acid-A receptor. Regulates the neurotransmitter gamma-aminobutyric acid (GABA) and plays a role in synaptic function^107^	Dysregulation of imprinting or issues in pairing of the homologous alleles^108^ via disruption of long-distanced chromosomal interactions^109–111^
		Decreased expression
*UBE3A*	Known for its role in Angelman syndrome	Loss of imprinting of one copy, and production of antisense RNA that binds to *UBE3A* and mRNA Prevents translation^112^
	Involved in the maintenance of synaptic plasticity and central for experience-dependent modifications in the brain^113^	
*GAD1*	Encodes an enzyme that catalyzes the decarboxylation of glutamate to GABA, the main inhibitory neurotransmitter	Increased hydroxymethylation and binding of MeCP2 (silencing) in *GAD1* promoters^53^
		Decreased expression
*EN2*	Encodes a homeodomain-containing protein and thought to play a role in controlling pattern formation during development of the central nervous system^114^	Hypermethylation and hydroxymethylation^54, 115^
		Increased *EN2* gene expression and translation
*RELN*	Regulates neuronal migration and positioning in the developing brain by way of cell–cell interactions	Enriched levels of 5-hmC at *RELN* promoter, increased binding of MeCP2 to 5-hmC
	Regulates synaptic plasticity by enhancement of the induction and maintenance of long-term potentiation	Reduced gene expression and translation^53, 116, 117^
*MECP2*	Encodes a methyl binding protein that binds methylated areas of DNA to silence genes. Has a role in synaptogenesis and long-term synaptic plasticity^118^	Several: decreased *MECP2* expression^119, 120^. Decreased MECP2hi protein cells associated with methylation of *MECP2* promoter^55, 121–123^
		Inability to define methylation and X-inactivation borders^56^
	Associated with Rett syndrome	Unlcear X-inactivation role^56, 124, 125^
		Other: *MECP2* regulation of other genes via epigenetics: recruitment of co-repressors, chromatin looping^126, 127^

*OXTR* oxytocin receptor, *GABRB3* gamma-aminobutyric acid-A receptor, *UBE3A* ubiquitin-protein ligase E3A gene, *GAD1* glutamate decarboxylase, *EN* engrailed 2, *RELN* Reelin, *MECP2* methyl CpG-binding protein 2

## Induced pluripotent stem cells and cerebral organoids: models for studying epigenetics in vitro

The neurobiology leading to ASD might already start early in the prenatal stages. Therefore, mimicking the prenatal development in vitro can give extremely valuable insight into the etiology of autism. In addition to that, the challenges in research using postmortem brain tissue warrants the utilization of other viable disease models, such as cell cultures, and in vivo recapitulation of brain development and related pathology on the basis of new, cutting edge technology involving induced pluripotent stem cells (iPSCs). IPSCs refer to somatic cells that have been reprogrammed to a pluripotent stage and then can be triggered to differentiate into all cell types in the human body, including neurons. The technology is relatively new; for the first time, it became clear about a decade ago that iPSCs could be generated via a set of transcription factors introduced to fibroblasts in vitro^60^. Other somatic cells have also been used in the generation of iPSCs, for example: keratinocytes^61^, epithelial bladder cells derived from urine^62^ as well as from hair follicles^63^. IPSCs and derived differentiated cells retain the genetic signature of the individual that they have been taken from and thus they represent a relevant disease model. IPSCs have also been utilized in ASD research^64^. The respective study found increased rates of proliferation of neural progenitor cells and neuron numbers in the ASD individual IPSCs who also had an increase of brain volume as seen on magnetic resonnce imaging. The increase in proliferation was due to dysregulation of the beta-catenin/BRN2 transcriptional cascade, and it seemed that the defects in neuronal networks could be rescued by insulin growth factor 1^64^.

Along with the advantages, there are also some additional challenges regarding iPSCs. Since it is a new technique, there is limited availability of “bio-banks”. Additionally, the cells themselves may present different phenotypes in two lines derived from the same person^65^. A possible solution to this issue could be the generation of several clones of each patient iPSC line but at additional time and cost. Reprogramming iPSCs has also been found to introduce mutations of the genomic DNA as well as exogenous reprogramming genes^66^. iPSCs sustain an epigenetic memory, essentially maintaining residual DNA methylation from the tissue of origin^67^. Most iPSCs are derived from differentiated somatic cells, namely, fibroblasts. This should be taken into account in the epigenetic studies of central nervous system in which iPSCs are used as a model and transdifferentiation is considered preferable to eliminate this challenge. There have also been reports on prolonged culturing affecting the transcriptomes and the phenotypes of cells^68^. Throughout the course of life, somatic cells (fibroblasts) accumulate somatic mutations. Individual clones from the same person could host different somatic mutations, as in the case of mitochondrial DNA in iPSC neurons^69^. The study reports that somatic mutations could randomly occur in individual cells and those mutations of mitochondrial DNA in iPSCs had a tendency to increase along with the age of the donor. Another study found that iPSC genomes potentially exhibit imperfectly rewired 3D folding linked to inaccurately reprogrammed gene expression^70^. How much this chromatin architecture is reorganized in the process of reprogramming remains uncertain. As it was mentioned before, a possible way around the disadvantages could be achieved by removing the iPSC step as the midway, i.e., transdifferentiation^71,72^. In this method, fibroblasts are directly converted into induced neural progenitor cells by way of timely restricted expression of the following genes: Sox2, Klf4, and c-Myc and limited and very strictly controlled expression of Oct4^73^. Another method based on forced expression of neurogenin 2 (Ngn2) turns skin fibroblasts into functional neurons^74^. Treatment of Ngn2-transduced fibroblasts with a mix of small molecules including SB431542 (a transforming growth factor-β inhibitor), CHIR99021 (a GSK3β inhibitor), and Noggin (a protein inhibitor of bone morphogenic protein signaling) increase conversion efficiency up to 85%^75^.

Two-dimensional (2D) cell cultures of iPSC and derived neurons offer easier environmental control, cell manipulation, and imaging; it is homogenic and has fair reproducibility^76,77^. Along with the advantages, there are also a set of disadvantages, including an inability to depict traits exhibited in vivo (e.g., gene expression), less compatibility with in vivo settings, as well as increased drug sensitivity (due to monolayer) and exposed surface.

The development of stem cell technology gave rise to the possibility of growing and differentiating iPSCs into small 3D structures called organoids. Cerebral organoids follow the in vivo timeline and can recapitulate the early (8–10 gestation week) to mid-fetal (22–24/35 gestation week) of human brain development. Depending on the presence or absence of growth factors and morphogens, different region-specific organoids can be formed such as the forebrain, midbrain, cortex, and hypothalamus. Moreover, brain organoids can exhibit a well-defined sub ventricular zone (SVZ) and six-layered cortical architecture typical for the human brain. However, the brain cortex and SVZ in the mid-fetal development are the main targets for modeling ASD. This makes cerebral organoids a valuable new tool for neurodevelopmental disorders. The organoid gene expression program is highly similar to human fetal tissue^78–84^.

The organoids more closely resemble the cells' natural environment, demonstrating cellular variety and intercellular communication, cell–matrix interactions, and complex transport systems of nutrients^85–91^. 2D monolayer cultures are not relevant in this aspect, leading them to be poorer determinants in drug and toxicity studies^92–95^. 3D cultures are a relevant model of cell migration, differentiation, and growth^86–91,96^. Furthermore, 3D cell cultures have a different gene expression compared to those grown in 2D^83,87,97^. There seems to be a dysregulation of morphology, response to environmental stress as well as cellular regulation in 2D cultures compared to 3D cultures^98^. This could limit the use of 2D cell culture in the search for epigenetic regulation in ASD since comparisons would be harder to make at the interface of gene–environment.

The 3D cell cultures offer several advantages such as: the cytoarchitecture is similar to that found in vivo, variety of cellular populations, organ specific functions, and niche-like environment. Carrying the specific genetic signature from the individual the cells were taken from, they can be utilized to compare the early brain structure and composition with ASD to that of their healthy family members as well as healthy controls. This means that researchers to a certain extent are able to model neurodevelopment in vitro and re-create the brain pathology related to ASD. This could also aid in elucidating the interface of gene expression levels and epigenetic changes^99–101^. The disadvantages related to this method include: diffusional transport limitations (oxygen, nutrients), culture-dependent alterations in gene expression, technical challenges in manipulation and imaging, standardization and reproducibility issues, as well as being time and labor consuming.

It should also be noted that the method still has an issue of “batch syndrome” in which different batches of organoids demonstrate significant variability in quality and brain regions they produce^102^. A very recent study analyzed 80,000 individual cells isolated from 31 human brain organoids. In addition to finding that organoids could generate a broad diversity of cells, they could also be developed over an extended period of time leading to the formation of dendritic spines as well as spontaneously active neuronal networks. Furthermore, this study also quantified variability among organoids and pointed to bioreactor-based batch effects as part of the explanation for the organoid-to-organoid variability^86^.

A recent study has conducted the first transcriptome and epigenome-wide sequencing of cerebral organoids by comparing them to human fetal brain, using genome-wide, base resolution DNA methylome and transcriptome sequencing^83^. Particularly, organoid differentiation was able to recapitulate the transcriptomic dynamics of early/middle fetal development. DNA methylation of cerebral organoids found a new kind of mCH enrichment (cytosine DNA methylation in non-CG contexts) that was indicative of transcriptional repression of the later brain in vivo. Hypermethylation of DNA methylation valleys, regions of low methylation of at least 5 kb in length (DMVs), served as a robust epigenomic signature of forebrain specification. Pervasive demethylation of pericentromeric repeats were found in human neural cultures that are not observed during fetal brain development. The last aspect points to the importance of genomic/epigenomic methods in the evaluation of in vitro-derived neuronal tissues vs. in vivo. The study came to the conclusion that the remodeling of mCG and mCH during the organoid differentiation resulted in an epigenomic state very similar to the human fetal brain^83^. It is for the moment not clear how epigenetic patterns of brain organoids could be verified by comparison with a human ES line and this should warrant further studies and be addressed.

**Fig. 2:**
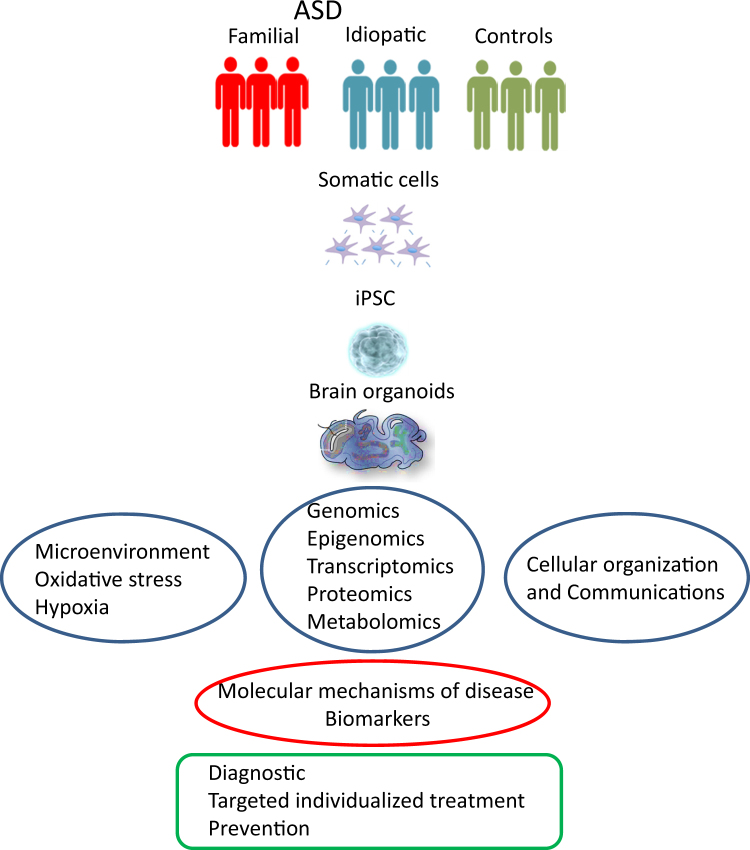
Schematic presentation of a brain organoid-focused study, with groups consisting of familial ASD cases, idiopathic cases, and a control group Somatic cells (for example, fibroblasts) can be obtained from the participants and reprogrammed into iPSC and furthermore into brain organoids specific for each individual. This could enable an in vitro multi-omics studies (genomics, transcriptomics, proteomics, epigenomics, metabolomics), oxidative stress, cellular organization, etc., hopefully, unveiling a molecular mechanism of the disorder that in the future can lead to better tools in diagnosis, prevention, and treatment

Studies concerned with iPSC epigenetic signatures could aid in the investigation of alterations in epigenetic regulation of gene expression in brain development. There is also added hope that this technique will in the future aid in drug discovery research (see Fig. 2).

## Conclusions

Several findings suggest that epigenetic alterations play a role in the development of ASD and further research is therefore warranted. This review points out that there is a necessity to carry out further EWAS studies. These should include larger numbers of patients who should be clinically well characterized, as well as investigate other, different epigenetic mechanisms as the majority of research has been focused on DNA methylation. The potential biomarkers, e.g., *C11ORF21* and *PRRT1*, identified in these studies should also be replicated. In addition, iPSCs and cerebral organoids could aid in bypassing the issue of obtaining sufficient postmortem brains, enabling a “disorder in a dish-setting”. Moreover, brain organoids are patient specific and can be utilized in the development of targeted personalized treatments. There are, however, some issues regarding this as well including, but not limited to, epigenetic memory of the tissue of origin in addition to issues regarding mutations and differing phenotypes from the same sample. For all the challenges posed, there are new findings that offer some valuable clues and that might guide the field forward, the most recent advance being the first epigenomic sequencing of cerebral organoids and a newly acquired understanding of how closely they could resemble the human brain. Studying organoids and the epigenetic alterations in the different brain structures during embryonal development might open up new avenues for new treatments targeting specific brain structures.
